# Assessing the implementation of healthy eating and physical activity policies and practices in Early Childhood Education and Care in New South Wales, Australia: A cross‐sectional study

**DOI:** 10.1002/hpja.917

**Published:** 2024-08-26

**Authors:** Ana Renda, Kathryn Reilly, Serene Yoong, Melanie Lum, Christophe Lecathelinais, Rebecca Hodder, Alice Grady

**Affiliations:** ^1^ School of Medicine and Public Health University of Newcastle Newcastle New South Wales Australia; ^2^ National Centre of Implementation Science University of Newcastle Newcastle New South Wales Australia; ^3^ Sydney Local Health District, Population Health Sydney New South Wales Australia; ^4^ Hunter Medical Research Institute New Lambton Heights New South Wales Australia; ^5^ Hunter New England Local Health District, Population Health Wallsend New South Wales Australia; ^6^ Global Centre for Preventive Health and Nutrition (GLOBE), Institute for Health Transformation Deakin University Burwood Victoria Australia

**Keywords:** childcare, healthy eating, implementation, obesity prevention, physical activity, policy, practice

## Abstract

**Issue Addressed:**

Australian children are not meeting the recommended physical activity (PA) and healthy eating (HE) guidelines. Health behaviour practices implemented in community settings such as early education services can improve child's health outcomes and are therefore key to help meet guidelines. This study aimed to measure the implementation of HE and PA policies and practices in Early Childhood Education and Care (ECEC) services in New South Wales (NSW), Australia, and to examine their association with service characteristics.

**Methods:**

A random sample of 1122 centre‐based ECEC services were invited to a cross‐sectional survey measuring HE and PA policy and practice implementation. Regression analyses were conducted to assess the relationship between the service characteristics and implementation of policies/practices.

**Results:**

In total, 565 ECEC services completed the survey. Results show that while some practices are implemented, the implementation of practices promoting HE and PA education is low. Practices related to educator training for HE (18%) and PA (13%) were poorly implemented. The implementation of practices such as ‘providing educator training around child PA’, ‘engaging families in activities to increase child PA’ and ‘encouraging supportive feeding practices’ were significantly higher in services located in major cities than regional/remote services. ‘Having a PA policy’ and the practice of ‘limiting the use of screen time’ was significantly higher in long day care services than in preschools. The implementation of ‘providing educator‐led PA’, ‘providing free play opportunities’ and ‘having a PA policy’ was significantly lower in private not‐for‐profit community managed services than in private for‐profit services.

**Conclusions:**

Implementation of various HE and PA policies and practices in regional/remote services, private not‐for‐profit services and preschools across NSW could be improved.

**So What?:**

Future research should be prioritised towards identifying factors influencing the implementation of these policies and practices to best tailor implementation support efforts for those who need it the most.

## 1 INTRODUCTION

Physical activity (PA) and an adequate dietary intake in children can decrease the risk of developing non‐communicable diseases, and these can track into adulthood.^1^, ^2^ In efforts to achieve optimal children's growth and development, international and national healthy eating (HE) and PA guidelines have been developed.^3^, ^4^, ^5^, ^6^, ^7^ However, children in Australia do not meet the recommended dietary intakes or levels of PA suggested by national guidelines.^8^, ^9^ Specifically, in 2017–2018, 94% of children aged 2–17 years did not meet the recommended intake for fruits and vegetables.^8^ Additionally, the latest data available on PA (2011–2012) indicate that 28% of children aged 2–5 years do not meet the recommended 180 minutes of PA per day.^9^

Given the large proportion of young children accessing Early Childhood Education and Care (ECEC) services globally, the World Health Organisation and the Institute Of Medicine^2^, ^10^, ^11^ recommend implementing HE and PA policies and practices in this setting. In Australia, centre‐based ECEC services are inclusive of preschools and long day care centres, which are attended by 40% of 0‐ to 2‐year‐olds and 84% of 3‐ to 5‐year‐olds,^12^ with more children attending private ECEC compared to government‐based services.^13^ ECEC providers are licenced through Australian Children's Education and Care Quality Authority (ACECQA) and are required to abide by the National Quality Framework and National Quality Standards, which support the implementation of HE and PA policies and practices.^14^, ^15^ For the purpose of this study, policy refers to a formal written document outlining the purpose, goal, scope, roles and responsibilities of the service in regards to HE and PA, and it mainly relates to private policy (i.e. created by an organisation for its internal use) rather than public policy (i.e. a law or regulation established by the government).^16^, ^17^ Whereas a practice is an action or initiative such as activities, programs or processes related to HE and PA of children within the service.^18^

Evidence from systematic and umbrella reviews indicates that HE and PA policies and practices recommended by international guidelines^19^ in the ECEC setting can improve HE and PA behaviours in children.^20^, ^21^, ^22^, ^23^, ^24^, ^25^, ^26^, ^27^, ^28^, ^29^ A recent Cochrane review found that practices delivered in ECEC settings, such as including healthier food and beverage options on the menu or implementing HE lessons, may improve children's diet quality and fruit consumption.^29^ A recent umbrella review synthesising PA practices found that interventions that provide opportunities for PA, educator PA training, PA‐promoting environment or family involvement can improve child PA levels.^28^ PA policy implementation has also shown to improve child light PA levels. For example, a pilot cluster randomised control trial in Canada examined the impact of the Childcare Physical Activity policy on children PA and sedentary time and found a positive effect on children's light PA levels at follow‐up (+1.07 min/h, an 11.16% increase; *p* = .0017).^30^ Another study in the United States showed that the implementation of a government nutrition policy significantly improved fruit (.8 points Healthy Eating Index; 95% confidence interval (CI) [.2, 1.5]; *p* = .01) and protein‐based foods intake (1.2 points Healthy Eating Index; 95% CI [.06, 2.3]; *p* = .04).^31^ As such, understanding the extent in which such practices are implemented routinely in ECECs is needed to support the ongoing refinement and development of child HE programs. For example, enhanced implementation support could be provided focusing on areas of low implementation.

Monitoring the implementation of HE and PA practices is needed to direct public health policies' efforts and inform allocation of resources. Since the World Health Organisation's recommendation of HE and PA practices in community settings, the implementation of such practices has been measured in the ECEC setting in different countries. However, evidence suggests that the implementation of such practices in ECEC settings internationally remains low. For example, in two states of the United States in 2017, an average of 7.4 out of 15 HE practices and 5.3 out of 10 PA practices were implemented in 428 centre‐based services.^32^ In New Zealand in 2016, only 35% of 237 ECEC services had a PA policy.^33^

The implementation of HE and PA practices in Australia is only partially known, due to the lack of up‐to‐date data, and a dearth of studies comprehensively and consistently assessing practices and the respective ECEC service characteristics. A longitudinal study, collecting data via computer‐assisted telephone interviews was conducted in New South Wales (NSW) (2006–2013) and indicated positive trends.^34^ However, it assessed a limited number of mixed HE and PA practices and service characteristics, hindering their ability to provide a comprehensive overview of the current implementation in Australia. Another cross‐sectional study conducted in 2017, measured via computer‐assisted telephone interviews the implementation of seven PA practices in a specific region (i.e. Hunter New England) of NSW and reported that only 39% of services have a PA policy and 8% of 309 services implemented all seven policies and practices.^35^ These findings show that the implementation of HE and/or PA practices has increased in the last few years; however, it remains variable. Further, neither of these studies examined associations between implementation and provider type, which has been shown to be associated with ECEC service quality ratings as assessed by ACECQA.^14^ While these Australian studies provide a high‐level snapshot of the implementation of HE and PA practices in ECECs, they only measure a few practices, show inconsistencies in the type of practice and/or policy reported, and were conducted over 5 years ago.^34^, ^35^ Therefore, the primary aim of this study is to describe the latest level of implementation of evidence‐based HE and PA policies and practice components in a representative sample of centre‐based ECEC services across NSW, Australia. For this study, level of implementation refers to the frequency, amount and type of components implemented of a policy or practice. As a secondary objective, we assess the extent of implementation of these practices, defined as the mean proportion of components implemented for each policy and practice. As a third objective, we assess the association between the extent of implementation of these practices (i.e. mean proportion of components) and service characteristics including service size, number of staff, service type, provider type, service socio‐economic status (SES) and location.

## 2 METHODS

This research received approval from HNE Human Research Ethics Committee (06/07/26/4.04 2019/ETH12353) and the University of Newcastle Human Research Ethics Committee (H‐2008‐0343). We report this study following the STrengthening the Reporting of OBservational studies in Epidemiology (STROBE) guidelines for cross‐sectional studies.^36^

### 2.1 Study design

We conducted an online or telephone cross‐sectional survey with centre‐based ECEC services in NSW, Australia, which includes long day care services and preschools.

### 2.2 Context

In NSW, Australia, a state‐wide obesity prevention program (i.e. Munch & Move) developed as part of the NSW Government's plan ‘Preventing Overweight and Obesity in Children, Young People and their families 2009‐2011’, has been implemented in preschools and in long day care services since 2008 and 2010, respectively. This program aims to develop supportive HE and PA environments^37^ in the ECEC setting. ECEC services are provided with support, resources and professional development to assist the implementation of the Munch & Move HE and PA promoting policies and practices. The Munch & Move program has undergone an evaluation which found a reduction in the prevalence of overweight/obesity, an improvement in the diet of preschoolers^38^ and in children's fundamental movement skills.^39^ Further description and evaluation of this program has been previously published.^37^, ^39^

### 2.3 Sample, setting and recruitment

We obtained NSW ECEC service contact details (*N* = 3748) from the publicly available ACECQA register.^14^ Services such as family day care, occasional care, outside of school hours care, Department of Education, temporarily closed (according to ACECQA), and those solely caring for children with special needs were deemed ineligible, as they tend to operate differently from the target sample. Services currently taking part in a national survey conducted by the research team were also ineligible to reduce participant burden. A final sample of 1122 services were randomly selected and invited to participate. This was determined to allow for recruitment of a sample of at least 500 services (accounting for 50% consent rate) of which 250 services would be assigned to respond to HE items and 250 to PA items to reduce survey length and participant burden. This number was estimated to allow the reporting of mean estimates with a precision of .12 *z*‐score units, using 95% CI.

We carried out recruitment of services using a staggered approach, emailing ~250 services per week for 5 weeks. We included in the invitation email, a link directing participants to the information statement and online survey. Nominated supervisors were the preferred respondents as they oversee the educational program and daily routines of the entire service. They are ‘responsible for the day‐to‐day management of an approved service’.^40^ If unavailable, they could nominate a delegate with knowledge of the overall service's HE and PA practice implementation (such as service director, second in charge or educational leader) to complete the survey. We sent a reminder email to services which did not complete the online survey within 1 week. If the survey was not completed within 2 weeks of the initial email, services received a telephone call from a trained interviewer and were invited to complete the survey by telephone. Trained interviewers used a pre‐approved script when conducting the telephone survey to reduce interviewer bias.^41^

### 2.4 Data collection, variables and measurement

We conducted the surveys between October 2021 and June 2022. Surveys took approximately 30 min to complete. We collected data using REDCap (Research Electronic Data Capture version 12.0.2), a secure web‐based application designed for data collection.^42^ For telephone surveys, services were randomly allocated to receive either HE or PA policies' and practices' items using block randomisation. For online surveys, simple randomisation between HE/PA items at a 1:1 ratio using the SURVEYSELECT procedure in statistical analysis software (SAS) was completed.

### 2.5 Service characteristics

Items previously used in other Australian surveys^34^, ^35^ were used to assess service characteristics including service type (long day care or preschool), days and hours of operation, number of staff, main position of respondent, number of children attending on any given day and number of children from non‐English speaking backgrounds. We retrieved service postcode and service provider type (e.g. state/territory and local government managed, private for‐profit, private not‐for‐profit community managed and private not‐for‐profit other organisations) from the ACECQA register.

### 2.6 HE and PA components, policies and practices

We designed a 51‐item survey measuring the implementation of evidence‐based HE and PA components. Survey items were based on a range of validated items and measures including those developed by the research team,^43^ the Nutrition and Physical Activity Self‐assessment for Child Care tool^44^ and the Environment and Policy Assessment and Observation‐Self Reported tool.^45^ For this study, we refer to each survey item as a practice component (e.g. ‘How often do the service staff sit and eat with the children?’ as one component of the practice ‘Staff sit with children during meals, and role model healthy behaviours’). It should be noted that some of these components are included in the Munch & Move program.^37^ To address the secondary aim, we grouped components into broader HE or PA policies or practices as reported in previous studies.^34^, ^35^, ^46^ Overall, eight overarching HE and seven PA policies and practices consisting of a total of 30 components and 21 components, respectively, were assessed (see Table 1).

**TABLE 1:** HE and PA policies and practices and their corresponding components.

Policy/practice	Component
*HE*	
Providing menu planning support	Service cook completed training in planning, preparing and serving nutritious meals and snacks.
	Planner/service cook use any nutrition guidelines to plan their menus.
	Planner/service cook receive any of the following support from your service to plan their menus.
Encouraging parents to pack healthy food from home and ensure foods bought from home meet nutrition written standards	Service provides a resource to families with recommendations for the types of food and drinks brought in lunch boxes from home.
	Resources refer to the Australian Dietary Guidelines.
	Frequency that the service observes children's lunch boxes to ensure that they are consistent with the Australian Dietary Guidelines.
	Frequency that the service provides feedback to families if a lunch box is not consistent with the Australian Dietary Guidelines.
Educators are trained in nutrition curriculum/education	In a typical year, the number of primary contact educators participate in professional development or training (e.g. face to face or online) which promotes HE for children.
	The service provides educators with any additional support or resources (e.g. peer‐support meetings with colleagues and newsletters) to increase HE for children in the past year.
Providing opportunities for nutrition education children	Service undertakes any planned HE education lessons (not including mealtime).
	Service undertakes any interactive, experiential HE activities (e.g. cooking lessons and discussions around food growing).
	Service intentionally exposes children to different vegetables as part of experiential learning.
	Service undertakes any play‐based HE activities? (e.g. use of puppets, mascot or other resources to communicate messages around HE).
	Service undertakes any HE‐themed special day (e.g. vegetable tasting day).
Staff sit with children during meals, and role model healthy behaviours	Frequency in which the service implements any programs or strategies to encourage children to role model HE to one another during mealtimes.
	Frequency the service staff sits and eats with the children.
	Frequency the service staff does not eat or drink unhealthy foods in front of the children.
	Frequency the service staff eats fruit in front of the children.
	Frequency the service staff eats vegetables in front of the children.
	Frequency the service staff enthusiastically role model eating healthy foods.
Encouraging healthy drink choices	Type of drink(s) service provides to children.
	Frequency the service implements strategies to encourage children to consume age‐appropriate beverages including water and milk.
Encouraging supportive feeding practices	Frequency the service staff encourage children to try a new or less preferred food.
	Frequency the service staff use children's preferred foods to encourage them to eat new or less preferred foods.
	Frequency the service staff does not use food to calm upset children or encourage appropriate behaviour.
	Frequency the service staff does not use food as a reward or incentive.
	Frequency the service staff does not require that children sit at the table until they finish their meal or snack.
Having a comprehensive HE policy	Service has a written nutrition policy.
	The nutrition policy includes any specific elements (e.g. sugar‐sweetened beverages are not available to children, or there is a separate sugar‐sweetened beverage policy, strategies are in place to ensure food is not used as a reward or incentive for children and professional development on child nutrition).
*PA*	
Scheduling more opportunities for outdoor free play	Services provide outdoor‐only free play per day.
	Services provide indoor‐only free play per day.
	Services provide indoor–outdoor free play per day.
Including opportunities for adult‐led, structured PA	Services provide structured (intentional) or educator‐led physical activities per day.
	Number of days in the past week the service provides an activity designed to intentionally teach and develop the various fundamental movement skills for children aged 3–6 years of age.
	Frequency educators lead energisers in the room with the most 3‐ to 6‐year‐olds.
	Frequency that educators led energisers per day.
Providing educator training on PA	Number of primary contact educators that participated in training (e.g. online or face‐to‐face) which promotes PA for children in the last year.
	Service provides educators with any additional support or resources to increase PA for children in the last year (e.g. peer‐support meetings with colleagues and newsletters).
Engaging families in activities to increase child PA	Service offered PA education to families in the form of family workshops or meetings in the last year.
	Service offered PA education to families in the form of handouts of newsletters articles, bulletin board topics, postings on the service's website, email, app, social media or other usual communication channels used by the service in the last years.
	In the last year, service sought feedback from families to contribute to the PA policies of your service in the last year.
Providing sufficient portable play equipment	Frequency service has portable play equipment available and in good condition for children to use.
	Staff believes there is sufficient portable play equipment for children to share.
Having a comprehensive PA policy	Service has a written policy, procedure or guideline encouraging PA
	PA policy, procedure or guideline includes specific elements such as a reference to the Australian 24‐Hour Movement Guidelines for the Early Years (birth to 5 years), PA is embedded in the daily curriculum through spontaneous and intentionally planned active, play time that is both child‐initiated and educator‐led, etc.
Limiting the use of screen time (less is best)	Small screen devices are used with children aged 2–5 years to gain knowledge or share information about a specific learning area or child's interest or to facilitate exploration of activity, dance or movement.
	Children under 2 years of age so not spend any time watching small screen devices
	Frequency of small screen devices on during meal or snack times
	Service has a written policy, procedure or guideline limiting child viewing of small screen devices (this could include TV, DVD, iPad or computer).
	Policy, procedure or guideline includes a reference to the Australian 24‐h Movement Guidelines for the Early Years (Birth to 5 Years), not using screens as a reward or to manage challenging behaviours, or that educators are encouraged to role model appropriate screen behaviours to the children.

Abbreviations: HE, healthy eating; PA, physical activity.

### 2.7 Statistical methods

We performed descriptive analysis (frequencies and proportions) to describe responses to the level of implementation for each HE and PA component, using SAS version 9.3.

To obtain the extent of implementation of each practice, we dichotomised service components into ‘implementing’ or ‘not implementing’ based on previous literature^19^, ^47^, ^48^ and consultation with research team members. To provide an estimation of the extent to which each policy/practice was implemented, practice mean proportions were calculated by summing the number of components within each practice deemed as ‘implementing’ and dividing by the total number of components assessed within the practice, then multiplying by 100 (reported as a percentage).

We conducted linear regressions to explore associations between the extent of implementation of HE and PA policies and practice components and service characteristics (service size, number of staff, service type, provider type, service socio‐economic status (SES) and location), controlling for service geographical location. We dichotomised SES into most disadvantaged or least disadvantaged according to the NSW median using service postcodes.^49^ We classified geographical location as major cities or inner/outer regional/remote Australia.^50^ We classified the service size as small (less than 80 children attending on any given day) or large (80 or more).^46^ To verify the presence of selection bias and test the representativeness of our sample, we performed chi‐squared tests to compare geographical location and SES among consenters (services that agreed to participate in the study) and non‐consenters (services that did not agree to participate).

Statistical significance was set at α < .05. Where services refused to answer an item, their response was treated as missing data and not included in the regression analysis. Items were only asked of a service if they were relevant to the individual service (e.g. if the service provided meals for children, items relating to parents packing food in lunch boxes were not asked).

## 3 RESULTS

### 3.1 Participants

Of the 1122 randomly selected services, 565 completed the survey (52% consent rate, 40 services ineligible and, 63% online). There were 266 (47%) services which responded to PA items and 299 (53%) services responded to HE items. A statistically significant smaller proportion of services located in major cities consented to participate in the survey (50% vs. 60%, 95% CI [1.14–2.05], *p* = .004) compared to non‐consenters. No statistically significant differences in service area SES were found between consenters and non‐consenters. The percentage of long day cares and preschools in our sample is similar to the state sample.^51^ Table 2 details responding service characteristics.

**TABLE 2:** ECEC service and respondent characteristics.

Characteristics (*N* = 565)	*n* (%)
Type of ECEC service	
Long day care	458 (81.06%)
Preschool	107 (18.94%)
Service operates 5 days per week	551 (97.52%)
Hours of operation per day (mean, SD)	10.19 (1.53)
Number of children attending on any given day (mean, SD)	47 (23.41)
Children from non‐English speaking background (mean, SD)	13 (20.26)
Total staff employed (full‐time + part‐time + casuals) (mean, SD)	16 (8.95)
Main position of respondent	
Nominated supervisor/educational leader	289 (51.14%)
Director/assistant director	226 (40%)
Educator or room leader	22 (3.89%)
Service owner	11 (1.95%)
Other (e.g. trainee, area manager, administrative officer, cook and multiple roles)	17 (3.01%)
Location	
Inner/outer regional/remote Australia	143 (25.31%)
Major cities	422 (74.69%)
SES (SEIFA 2016)	
Most disadvantage	238 (42.12%)
Least disadvantage	327 (57.88%)
Service provider type	
Private for‐profit	341 (60.35%)
Private not‐for‐profit community managed	132 (23.36%)
Private not‐for‐profit other organisations	48 (8.50%)
State/territory and local government managed (i.e. local council managed)	44 (7.79%)
Survey method	
Online	355 (62.83%)
Telephone	210 (37.17%)

Abbreviations: ECEC, Early Childhood Education and Care; HE, healthy eating; PA, physical activity.

### 3.2 Implementation of components of HE policies and practices

In services where food is prepared on‐site, 76% of services (*n* = 163) reported that cooks/chefs have completed training in planning, preparing and serving nutritious meals and snacks (Table 3). In services where families provide food in lunch boxes (*n* = 101), 97% of services reported providing a resource with recommendations for the types of food and drinks to pack to all families. Less than 30% of services reported all primary contact educators participate in professional development promoting HE for children (17.6%), with approximately one‐third (29%) reporting to provide planned HE education lessons weekly. During mealtimes, 93% of services reported that staff sit and eat with children daily. Lastly, 98% of services reported having a written nutrition policy.

**TABLE 3:** Level of implementation of components of HE and PA policy and practice items.

Policy/practice	Component	Responses	*n* (%)
HE			
Providing menu planning support	Has your service cook completed training in planning, preparing and serving nutritious meals and snacks? (*n* = 163)	Yes^a^	123 (75.46%)
		No	28 (17.18%)
		Do not know	12 (7.36%)
	Does the planner/service cook use any nutrition guidelines to plan their menus? (*n* = 158)	Yes^a^	148 (93.67%)
		No	1 (.63%)
		Do not know	9 (5.70%)
	Does the planner or service cook receive any of the following support from your service to plan their menus?^b^ (*n* = 163)	Support from external experts (e.g. health promotion team and dietitian)^a^	105 (64.42%)
		Network or professional networking opportunities^a^	71 (43.56%)
		Peer‐learning or mentoring^a^	58 (35.58%)
		Written resources (e.g. recipes and books)^a^	113 (69.33%)
		Updated guidelines/nutrition recommendations^a^	120 (73.62%)
		Feedback from families^a^	5 (1.78%)
		Other (e.g. Munch and Move and experience working in other industries)^a^	14 (8.59%)
Encouraging parents to pack healthy food from home and ensure foods brought from home meet nutrition written standards	Does your service provide a resource to families with recommendations for the types of food and drinks brought in lunch boxes from home? (*n* = 101)	Yes, service provides this to all parents^a^	98 (97.03%)
		No, service does not provide any resources to parents	1 (.99%)
		Do not know	2 (1.98%)
	Do these resources refer to the Australian Dietary Guidelines? (*n* = 97)	Yes^a^	90 (92.78%)
		No	4 (4.12%)
		Do not know	3 (3.09%)
	How often does your service observe children's lunch boxes to ensure that they are consistent with the Australian Dietary Guidelines? (*n* = 97)	Every day^a^	83 (83.84%)
		Three to four times per week	4 (4.04%)
		One to two times per week	1 (1.01%)
		Service observes, but without reference to the Australia Dietary Guidelines	9 (9.09%)
		Service does not observe	1 (1.01%)
		Do not know	1 (1.01%)
	If a lunch box is not consistent with the Australian Dietary Guidelines, how often does the service provide feedback to families? (*n* = 89)	Never	2 (2.25%)
		Daily^a^	38 (42.70%)
		At least weekly^a^	13 (14.61%)
		At least monthly^a^	24 (26.97%)
		Quarterly (each term) or less often^a^	10 (11.24%)
		Not applicable. Lunch box is always consistent	2 (2.25%)
Educators are trained in nutrition curriculum/education	In a typical year, how many of your primary contact educators participate in professional development or training which promotes HE for children? This may include face to face or online training (*n* = 265)	All^a^	47 (17.74%)
		Almost all^a^	32 (12.08%)
		About half	43 (16.23%)
		Less than half	88 (33.21%)
		None	38 (14.34%)
		Do not know	17 (6.42%)
	In the past year, did the service provide educators with any of the following additional support or resources to increase HE for children?^b^ (*n* = 242)	Peer‐support meetings with colleagues^a^	126 (52.07%)
		Refresher training^a^	132 (54.55%)
		Newsletters^a^	158 (65.29%)
		Feedback from your service or other organisations^a^	101 (41.74%)
		Telephone support^a^	29 (11.98%)
		Munch & Move^a^	6 (2.01%)
		Other (e.g. resources, newsletters, webinars; family feedback, Facebook group, etc.)^a^	17 (7.02%)
		Do not know	12 (5.66%)
Providing opportunities for nutrition education children	Does your service undertake any planned HE education lessons (not including mealtime)? (*n* = 266)	Yes daily^a^	51 (19.17%)
		Yes weekly^a^	78 (29.32%)
		Yes monthly	82 (30.83%)
		Yes, less than monthly	40 (15.04%)
		No, not at all	8 (3.01%)
		Do not know	7 (2.63%)
	Does your service undertake any interactive, experiential HE activities (e.g. cooking lessons and discussions around food growing)? (*n* = 265)	Yes daily^a^	41 (15.47%)
		Yes weekly^a^	114 (43.02%)
		Yes monthly	77 (29.06%)
		Yes, less than monthly	29 (10.94%)
		No, not at all	2 (.75%)
		Do not know	2 (.75%)
	Does your service intentionally expose children to different vegetables as part of experiential learning? (*n* = 266)	Yes daily^a^	48 (18.05%)
		Yes weekly^a^	91 (34.21%)
		Yes monthly	75 (28.20%)
		Yes, less than monthly	37 (13.91%)
		No, not at all	10 (3.76%)
		Do not know	5 (1.88%)
	Does your service undertake any play‐based HE activities? (e.g. use of puppets, mascot or other resources to communicate messages around HE) (*n* = 266)	Yes daily^a^	47 (17.67%)
		Yes weekly^a^	79 (29.70%)
		Yes monthly	53 (19.92%)
		Yes, less than monthly	54 (20.30%)
		No, not at all	25 (9.40%)
		Do not know	8 (3.01%)
	Does your service undertake any HE‐themed special days? (e.g. vegetable tasting day) (*n* = 265)	Yes daily^a^	2 (.75%)
		Yes weekly^a^	16 (6.04%)
		Yes monthly	62 (23.4%)
		Yes, less than monthly	62 (23.4%)
		No, not at all	112 (42.26%)
		Do not know	11 (4.15%)
Staff sit with children during meals, and role model healthy behaviours	How often does your service implement any programs or strategies to encourage children to role model HE to one another during mealtimes? (*n* = 298)	Daily (or every day the service is open)^a^	229 (76.85%)
		At least weekly	32 (10.74%)
		At least monthly	16 (5.37%)
		Less than monthly	6 (2.01%)
		Never	9 (3.02%)
		Do not know	6 (2.01%)
	How often do the service staff sit and eat with the children? (*n* = 264)	Daily (or every day the service is open)^a^	245 (92.80%)
		At least weekly	12 (4.55%)
		At least monthly	1 (.38%)
		Less than monthly	2 (.76%)
		Never	4 (1.52%)
		Do not know	0
	How often do the service staff eat or drink unhealthy foods in front of the children? (*n* = 263)	Daily (or every day the service is open)^a^	10 (3.8%)
		At least weekly	3 (1.14%)
		At least monthly	4 (1.52%)
		Less than monthly	22 (8.37%)
		Never	218 (82.89%)
		Do not know	6 (2.28%)
	How often do the service staff eat fruit in front of the children? (*n* = 264)	Daily (or every day the service is open)^a^	236 (89.39%)
		At least weekly	18 (6.82%)
		At least monthly	1 (.38%)
		Less than monthly	4 (1.52%)
		Never	2 (.76%)
		Do not know	3 (1.14%)
	How often do the service staff eat vegetables in front of the children? (*n* = 264)	Daily (or every day the service is open)^a^	218 (82.58%)
		At least weekly	26 (9.85%)
		At least monthly	6 (2.27%)
		Less than monthly	4 (1.52%)
		Never	2 (.76%)
		Do not know	8 (3.03%)
	How often do service staff enthusiastically role model eating healthy foods? (*n* = 264)	Daily (or every day the service is open)^a^	236 (89.39%)
		At least weekly	19 (7.20%)
		At least monthly	3 (1.14%)
		Less than monthly	1 (.38%)
		Never	2 (.76%)
		Do not know	3 (1.14%)
Encouraging healthy drink choices	What drink(s) does your service provide to children?^b^ (*n* = 298)	Fruit juice or fruit drink including 100% fruit juice	2 (.67%)
		Cordial	0
		Water^a^	296 (99.33%)
		Full‐cream milk^a^	166 (55.70%)
		Reduced fat milk (including lite or low‐fat milk)^a^	146 (48.99%)
		Flavoured milk	1 (.34%)
		Soft drink	0
		Alternative milk types (e.g. soy milk and oat milk)	10 (3.34%)
		Other (e.g. smoothies and formula)	18 (6.04%)
		No drinks provided	2 (.67%)
		Do not know	0
	How often does your service implement strategies to encourage children to consume age‐appropriate beverages including water and milk? (*n* = 299)	Daily (or every day the service is open)^a^	292 (97.66%)
		2–4 days per week	3 (1.00%)
		Once per week	1 (.33%)
		Less than once per week	1 (.33%)
		Never	1 (.33%)
		Do not know	1 (.33%)
Encouraging supportive feeding practices	How often do the service staff encourage children to try a new or less preferred food? (*n* = 264)	Every time a new or less preferred food is provided in the lunch box or on menu^a^	223 (84.47%)
		At least weekly	22 (8.33%)
		At least monthly	8 (3.03%)
		Less than monthly	8 (3.03%)
		Never	2 (.76%)
		Do not know	1 (.38%)
	How often do service staff use children's preferred foods to encourage them to eat new or less‐preferred foods? (*n* = 261)	Every time a new or less preferred food is provided in the lunch box or on menu^a^	153 (58.62%)
		At least weekly	34 (13.03%)
		At least monthly	9 (3.45%)
		Less than monthly	9 (3.45%)
		Never	42 (16.09%)
		Do not know	14 (5.36%)
	How often do service staff use food to calm upset children or encourage appropriate behaviour? (*n* = 264)	Daily (or every day the service is open)	7 (2.65%)
		At least weekly	5 (1.89%)
		At least monthly	2 (.76%)
		Less than monthly	14 (5.30%)
		Never^a^	229 (86.74%)
		Do not know	7 (2.65%)
	How often do the service staff use food as a reward or incentive? (*n* = 264)	Daily (or every day the service is open)	4 (1.52%)
		At least weekly	6 (2.27%)
		At least monthly	5 (1.89%)
		Less than monthly	14 (5.30%)
		Never^a^	232 (87.88%)
		Do not know	3 (1.14%)
	How often do service staff require that children sit at the table until they finish their meal or snack? (*n* = 260)	Daily (or every day the service is open)	97 (37.31%)
		At least weekly	5 (1.92%)
		At least monthly	2 (.77%)
		Less than monthly	5 (1.92%)
		Never^a^	140 (53.85%)
		Do not know	11 (4.23%)
Having a comprehensive health eating policy	Does your service have a written nutrition policy? (*n* = 296)	Yes^a^	289 (97.64%)
		No	2 (.68%)
		Do not know	5 (1.69%)
	Does your nutrition policy include any of the following elements^b^ (*n* = 289)	Strategies are in place to ensure that foods provided by families is consistent with the Australian Dietary Guidelines (lunch box services only)^a^	129 (55.36%)
		Food provided by your service is consistent with the Australian Dietary Guidelines (menu services only)^a^	196 (67.82%)
		Sugar‐sweetened beverages are not available to children, or there is a separate sugar‐sweetened beverage policy^a^	226 (78.20%)
		Strategies are in place to ensure food is not used as a reward or incentive for children^a^	224 (77.51%)
		Educators role model healthy food and drink choices^a^	263 (91.00%)
		Creating healthy mealtime environments^a^	263 (91.00%)
		Educator practices to encourage HE^a^	259 (89.62%)
		Planned and informal nutrition education for children^a^	243 (84.08%)
		Professional development on child nutrition^a^	188 (65.05%)
		Guidelines for foods offered during holidays and celebrations^a^	154 (53.29%)
		None of the above	0
	Does your beverage/drinks policy include any of the following elements^b^ (*n* = 298)	Supporting water as a drink for children aged 3–5 years^a^	252 (84.56%)
		Drinks provided/allowed by your service are consistent with the Australian Dietary Guidelines or Caring for Children Guidelines and age appropriate^a^	200 (67.11%)
		Sugar‐sweetened beverages are not available to children^a^	181 (60.74%)
		Educators role model healthy drink choices^a^	217 (72.82%)
		None of the above	1 (.34%)
		Not applicable, there is no beverage/drinks policy	30 (10.07%)
*PA*			
Scheduling more opportunities for outdoor free play	Of the total time provided for child‐initiated free play, how much of that time was for outdoor‐only free play per day? (*n* = 263)	if >60 min^a^	182 (69%)
	Of the total time provided for child‐initiated free play, how much of that time was for indoor–outdoor free play per day? (*n* = 263)	≥1 min^a^	216 (82%)
	Of the total time provided for child‐initiated free play, how much of that time was for indoor‐only free play per day? (*n* = 263)	≥1 min^a^	187 (71%)
Including opportunities for adult‐led, structured PA	Services providing structured (intentional) or educator‐led physical activities per day? (*n* = 265)	if >60 min^a^	205 (77.36%)
	Thinking about the last week, how many days a week did your service provide an activity designed to intentionally teach and develop the various fundamental movement skills for children aged 3–6 years of age? (*n* = 263)	Never	4 (1.52%)
		One day	7 (2.66%)
		Two days	9 (3.42%)
		Three days	16 (6.08%)
		Four days	11 (4.18%)
		Daily (or every day the service is open)^a^	214 (81.37%)
		Do not know	2 (.76%)
	Thinking about the last week, how often did educators lead energisers in the room with the most 3‐ to 6‐year‐olds? (*n* = 264)	Never	10 (3.79%)
		One day	4 (1.52%)
		Two days	9 (3.41%)
		Three days	23 (8.71%)
		Four days	14 (5.30%)
		Daily (or every day the service is open)^a^	196 (74.24%)
		Do not know	8 (3.03%)
	On a day that educators led energisers, how often did this occur? (*n* = 246)	Once	67 (27.24%)
		Twice	85 (34.55%)
		Three times^a^	53 (21.54%)
		More than three times^a^	36 (14.63%)
		Do not know	5 (2.03%)
Providing educator training on PA	In the past year, how many of your primary contact educators participated in training which promotes PA for children? This can include online or face‐to‐face training (*n* = 231)	All^a^	31 (13.42%)
		Almost all^a^	15 (6.49%)
		About half	31 (13.42%)
		Less than half	67 (29.00%)
		None	76 (32.90%)
		Do not know	11 (4.76%)
	In the past year, did your service provide educators with any of the following additional support or resources to increase PA for children?^b^ (*n* = 262)	Peer‐support meetings with colleagues^a^	131 (50.00%)
		Refresher training^a^	104 (39.69%)
		Newsletters^a^	132 (50.38%)
		Feedback from your service or other organisation^a^	87 (33.21%)
		Telephone support^a^	26 (11.07%)
		Munch & Move^a^	5 (1.89%)
		Other: meetings, equipment, grow fit program, head office support team and WhatsApp group between educators^a^	29 (11.29%)
		None	30 (11.45%)
Engaging families in activities to increase child PA	In the past year, did you offer PA education to families in the form of family workshops or meetings? (*n* = 261)	Yes, to all parent/families^a^	35 (13.41%)
		Yes, to parent/families of 3–6‐year‐olds only	3 (1.15%)
		No	210 (80.46%)
		Do not Don't know	13 (4.98%)
	In the last year, did you offer PA education to families in the form of handouts newsletters articles, bulletin board topics, postings on the service's website, email, app, social media or other usual communication channels used by the service? (*n* = 262)	Yes, to all parent/families^a^	183 (69.85%)
		Yes, to parent/families of 3‐ to 6‐year‐olds only	15 (5.73%)
		No	54 (20.61%)
		Do not know	10 (3.82%)
	In the last year, did your service seek feedback from families to contribute to the PA policies of your service? (*n* = 229)	Yes^a^	107 (46.72%)
		No	108 (47.16%)
		Do not know	14 (6.11%)
Providing sufficient portable play equipment	How often does your service have portable play equipment available and in good condition for children to use? (*n* = 266)	Always^a^	225 (84.59%)
		Often	30 (11.28%)
		Sometimes	9 (3.38%)
		Never	1 (.38%)
		Do not know	1 (.38%)
	In your opinion, is there sufficient portable play equipment for children to share? (*n* = 266)	Yes^a^	227 (85.34%)
		No	30 (11.28%)
		Don't know	7 (2.63%)
Having a comprehensive PA policy	Does your service have a written policy, procedure or guideline encouraging PA? (*n* = 260)	Yes, independent policy, guideline or procedure^a^	126 (48.46%)
		Yes, integrated within another policy, guideline or procedure^a^	105 (40.38%)
		No	17 (6.54%)
		Do not know	12 (4.62%)
	Does your PA policy, procedure or guideline include the following elements?^b^ (*n* = 231)	Reference to the Australian 24‐Hour Movement Guidelines for the Early Years (birth to 5 years)^a^	146 (63.20%)
		PA is embedded in the daily curriculum through spontaneous and intentionally planned active, play time that is both child‐initiated and educator‐led^a^	204 (88.31%)
		Educators actively role model appropriate PA behaviours to children^a^	200 (86.58%)
		Professional development on children's PA^a^	141 (61.04%)
		Education for families on children's PA^a^	133 (57.58%)
		None of the above	3 (1.30%)
Limiting the use of screen time (less is best)	For which purpose(s) do children aged 2–5 years spend time watching small screen devices? Please select all that apply^b^ (*n* = 260)	Not applicable (e.g. service does not use/have small screen devices)^a^	79 (30.38%)
		To gain knowledge or share information about a specific learning area or child's interest^a^	167 (64.23%)
		For child amusement, enjoyment or entertainment	10 (3.85%)
		To facilitate exploration of activity, dance or movement^a^	97 (37.31%)
		For down time or quiet time	18 (6.92%)
	Do children under 2 years of age spend any time watching small screen devices? (*n* = 112)	Yes	9 (8.04%)
		No^a^	100 (90.18%)
		Not applicable (e.g. service does not use/have small screen devices or no children less than 2 years of age)^a^	2 (1.79%)
	How often are small screen devices on during meal or snack times? (*n* = 182)	Daily (or every day the service is open)	6 (3.30%)
		At least weekly	13 (7.14%)
		At least monthly	2 (1.10%)
		Less than monthly	8 (4.40%)
		Never^a^	151 (82.97%)
		Do not Don't know	2 (1.10%)
	Does your service have a written policy, procedure or guideline limiting child viewing of small screen devices? (This could include TV, DVD, iPad or computer) (*n* = 261)	Yes^a^	191 (79.18%)
		No	38 (14.56%)
		Not applicable, service does not use/have small screen devices^a^	23 (8.81%)
		Do not know	9 (3.45%)
	Does your policy, procedure or guideline include the following elements^b^ (*n* = 173)	Reference to the Australian 24‐Hour Movement Guidelines for the Early Years (birth to 5 Years)^a^	126 (65.97%)
		Not using screens as a reward or to manage challenging behaviours^a^	136 (71.20%)
		Educators are encouraged to role model appropriate screen behaviours to the children^a^	143 (74.87%)
		None of the above	13 (6.81%)

Abbreviations: HE, healthy eating; PA, physical activity.

^a^ Response option that deems component to be ‘implemented’.

^b^ For multiple response items, any one of the denoted response options was classified as ‘implementing’.

### 3.3 Implementation of components of PA policies and practices

Sixty‐nine per cent of services reported providing outdoor‐only free play and 71% providing indoor–outdoor free play each day (Table 3). Eighty‐two per cent of services reported providing daily activities designed to intentionally teach and develop fundamental movement skills and 84% reported that portable play equipment is always available and in good condition for children to use. Only 13% of services reported all primary contact educators have participated in training which promotes PA for children. In the last year, only 21% of services offered a workshop or meeting to families that included PA education. Most services reported having a written PA policy (90%) and a screen time policy (75%).

### 3.4 Extent of HE and PA policy and practice components implemented

Services implemented, on average, 98.65% of components of the HE practice ‘encouraging healthy drink choices’ and 93.24% of components of ‘having a comprehensive nutrition policy’ (Table 4). Services also implemented, on average, 42.65% of components for ‘providing opportunities for nutrition education for children’ and 63.41% of components for ‘educator trained in nutrition curriculum/education’.

**TABLE 4:** Extent of implementation of HE and PA policies and practices components.

Policy/practice	Extent of implementation of policies/practices
	Mean (SD)
*HE*	
Providing menu planning support (*n* = 163)	88.14 (19.49)
Encouraging parents to pack healthy food from home and ensure foods bought from home meet nutrition written standards (*n* = 101)	88.70 (23.91)
Educators are trained in nutrition curriculum/education (*n* = 297)	63.41 (31.64)
Providing opportunities for nutrition education children (*n* = 266)	42.65 (31.22)
Staff sit with children during meals, and role model healthy behaviours (*n* = 264)	72.40 (19.43)
Encouraging healthy drink choices (*n* = 296)	98.65 (8.12)
Encouraging supportive feeding practices (*n* = 264)	74.55 (21.52)
Having a comprehensive HE Policy (*n* = 296)	93.24 (17.12)
*PA*	
Scheduling more opportunities for outdoor free play (*n* = 263)	74.14 (25.37)
Including opportunities for adult‐led, structured PA (*n* = 265)	66.67 (25.41)
Providing educator training on PA (*n* = 231)	51.30 (30.19)
Engaging families in activities to increase child PA (*n* = 261)	41.51 (29.80)
Providing sufficient portable play equipment (*n* = 266)	84.96 (29.13)
Having a comprehensive PA policy (*n* = 260)	85.77 (32.72)
Limiting the use of screen time (less is best) (*n* = 261)	84.66 (21.12)

Abbreviations: HE, healthy eating; PA, physical activity.

Services reported to implement, on average, 85.77% of components of the PA practice ‘having a comprehensive PA policy’ and 84.96% of components of ‘providing sufficient portable play equipment’ (Table 4). Services also implemented, on average, 41.51% of components for ‘engaging families in activities to increase child PA’ and 51.30% of components for ‘providing educator training on PA’ (51.30%).

### 3.5 Associations between service characteristics and mean proportion of practice implementation

#### 3.5.1 HE policies and practices

‘Encouraging supportive feeding practices’ was implemented significantly higher in services located in major cities compared to services located in remote areas (*β* = 9.10, 95% CI [3.26–14.94], *p* = .002) (Table 5). ‘Promoting HE education lessons to children’ was implemented at a significantly lower extent in state/territory and local government managed compared to private for‐profit services (*β* = −17.32, 95% CI [−30.99–(−3.66)], *p* = .013). Lastly, ‘having a comprehensive nutrition policy’ was implemented at a significantly lower extent in private not‐for‐profit other organisation services compared to private for‐profit services (*β* = −8.82, 95% CI [−16.14–(−1.50)], *p* = .018). No statistically significant associations were found between the implementation of HE practices and the type of service, socio‐economic status, service size or total number of staff employed.

**TABLE 5:** Associations between service characteristics and implementation of HE policies and practices.

	Type of service		Remoteness		Socio‐economic status		Provider type						Service size		Total number of staff employed	
	Long day care vs. preschools		Major cities vs. inner/outer regional/remote Australia		Most disadvantaged vs. least disadvantaged		Private not‐for‐profit community managed vs. private for‐profit		Private not‐for‐profit other organisations vs. private for‐profit		State/territory and local government managed vs. private for‐profit		Large (≥80) vs. small (<80)			
	Estimate, [95% CI]	*p*	Estimate, [95% CI]	*p*	Estimate, [95% CI]	*p*	Estimate, [95% CI]	*p*	Estimate, [95% CI]	*p*	Estimate, [95% CI]	*p*	Estimate, [95% CI]	*p*	Estimate, [95% CI]	*p*
Accessing menu planning support^a^ (*n* = 163)	4.92 [−11.17–21.02]	.55	−.99 [−8.07–6.10]	.78	−2.31 [−8.93–4.31]	.49	4.14 [−6.50–14.77]	.44	6.96 [−2.18–16.10]	.13	−1.56 [−11.89–8.78]	.77	.44 [−7.85–8.73]	.92	.04 [−.29–.37]	.82
Encourage parents to pack healthy food from home and ensure foods bought from home meet nutrition written standards^a^ (*n* = 101)	−.31 [−10.04–9.41]	.95	1.57 [−8.22–11.35]	.75	.03 [−10.89–10.95]	1.00	4.36 [−5.65–14.36]	.39	12.26 [−22.22–46.75]	.48	−12.22 [−30.58–6.13]	.19	NR	NR	−.32 [−1.34–.70]	.53
Educators are trained in nutrition curriculum/education^a^ (*n* = 297)	−.71 [−9.98–8.56]	.88	.22 [−8.00–8.45]	.96	5.88 [−2.09–13.84]	.15	−1.07 [−10.08–7.93]	.81	8.65 [−4.71–22.00]	.20	9.95 [−3.37–23.28]	.14	−1.52 [−13.76–10.72]	.81	.15 [−.26–.56]	.46
Providing opportunities for nutrition education^a^ (*n* = 266)	3.76 [−6.15–13.67]	.46	−1.12 [−9.73–7.50]	.80	2.06 [−6.59–10.70]	.64	−3.21 [−12.60–6.18]	.50	−2.45 [−16.39–11.49]	.73	**−17.32** * **[−30.99–3.66]**	**.013** *	6.54 [−6.19–19.27]	.31	0.34 [−.08–.77]	.11
Staff sit with children during meals, and role model healthy behaviours^a^ (*n* = 264)	.47 [−5.71–6.64]	.88	1.87 [−3.49–7.24]	.49	−3.36 [−8.73–2.01]	.22	−.89 [−6.80–5.01]	.77	3.38 [−5.37–12.14]	.45	−1.92 [−10.68–6.84]	.67	−4.38 [−12.30–3.54]	.28	−0.05 [−.32–.21]	.69
Encouraging supportive feeding practices^a^ (*n* = 264)	−2.15 [−8.86–4.57]	.53	**9.10** * **[3.26–14.94]**	**.002** *	−4.26 [−10.10–1.58]	.15	6.02 [−0.37–12.42]	.06	4.38 [−5.10–13.87]	.36	3.48 [−6.01–12.96]	.47	4.51 [−4.11–13.14]	.30	0.12 [−0.17–0.41]	.43
Encouraging healthy drink choices^a^ (*n* = 296)	0.55 [−1.81–2.92]	.65	−1.83 [−3.94–0.28]	.09	−0.46 [−2.51–1.60]	.66	−0.69 [−3.01–1.63]	.56	−1.19 [−4.62–2.23]	.49	−1.12 [−4.54–2.31]	.52	−0.24 [−3.36–2.89]	.88	0.03 [−0.08–0.13]	.60
Having a comprehensive HE policy^a^ (*n* = 296)	−2.47 [−7.47–2.54]	.33	1.27 [−3.18–5.72]	.57	−3.75 [−8.05–0.56]	.09	−0.03 [−4.87–4.81]	.99	**−8.82** * **[−16.14–1.50]**	**.018** *	3.74 [−3.43–10.90]	.31	3.56 [−3.05–10.17]	.29	0.12 [−0.11–0.34]	.30

*Note*: NR, not reported as services where parents provided food had less than 80 children present on any given day.

Abbreviations: HE, healthy eating; PA, physical activity.

^a^ Controlled for remoteness.

* Significant at *p* < .05.

#### 3.5.2 PA policies and practices

‘Having a comprehensive PA policy’ and ‘limiting screen time use’ was implemented at a significantly higher extent in long day cares, compared to preschools (*β* = 11.86, 95% CI [1.64–22.07], *p* = .023; *β* = 11.77, 95% CI [5.22–18.32], *p* = <.001 respectively) (Table 6). ‘Providing educators with annual training opportunities and ongoing support’ and ‘engaging families in activities to increase child PA’ were implemented at a significantly higher extent in services located within major cities than in services in remote areas (*β* = 12.48, 95% CI [2.79–22.17], *p* = .012; and *β* = 12.93, 95% CI [4.67–21.2], *p* = .002). ‘Providing sufficient portable play equipment’ was implemented at a significantly higher extent in large services (>80 children present) than in smaller services (<80 children present) (*β* = −15.47, 95% CI [−27.00–(−3.95)], *p* = .009). The total number of educators was negatively associated with ‘engaging families in activities to increase child physical activity’ (*β* = −.43, 95% CI [−.83–(−.09)], *p* = .032), but positively associated with ‘limiting screen time use’ (*β* = .44, 95% CI [.16–.72], *p* = .003).

**TABLE 6:** Associations between service characteristics and implementation of PA policies and practices.

	Type of service		Remoteness		Socio‐economic status		Provider type						Service size		Total number of staff employed	
	Long day care vs. preschool		Major cities vs. inner/outer regional/remote Australia		Most disadvantaged vs. least disadvantaged		Private not‐for‐profit community managed vs. private for‐profit		Private not‐for‐profit other organisations vs. private for‐profit		State/territory and local government managed vs. private for‐profit		Large (≥80) vs. small (<80)			
	Estimate, [95% CI]	*p*	Estimate, [95% CI]	*p*	Estimate, [95% CI]	*p*	Estimate, [95% CI]	*p*	Estimate, [95% CI]	*p*	Estimate, [95% CI]	*p*	Estimate, [95% CI]	*p*	Estimate, [95% CI]	*p*
Scheduling more opportunities for outdoor free play^a^ (*n* = 263)	5.35 [−2.66–13.35]	.19	−6.64 [−13.78–.51]	.07	−.34 [−6.95–6.27]	.92	**−14.05** * **[−21.52–6.58]**	**<.001** *	−3.64 [−14.51–7.23]	.51	.23 [−11.59–12.06]	.97	7.86 [−2.27–18.00]	.13	.16 [−.18–.50]	.36
Include opportunities for adult‐led, structured PA^a^ (*n* = 265)	7.77 [−.19–15.73]	.056	−.52 [−7.71–6.68]	.89	−2.05 [−8.67–4.58]	.54	**−9.62** * **[−17.06‐(−2.18)]**	**.011** *	**−12.78** * **[−23.76‐(−1.80)]**	**.023** *	**−14.41** * **[−26.35–2.47]**	**.018** *	−7.22 [−17.43–3.00]	.17	−.19 [−.53–.14]	.26
Providing educator training on PA^a^ (*n* = 231)	.59 [−9.38–10.57]	.91	**12.48** * **[2.79–22.17]**	**.012** *	7.17 [−1.25–15.60]	.09	−2.70 [−12.39–6.99]	.58	−3.29 [−17.42–10.85]	.65	−5.82 [−20.28–8.63]	.43	−5.46 [−18.44–7.53]	.41	−.18 [−.61–.26]	.42
Engaging families in activities to increase child PA^a^ (*n* = 261)	7.98 [−1.20–17.16]	.09	**12.93** * **[4.67–21.20]**	**.002** *	4.61 [−3.11–12.33]	.24	−5.18 [−13.89–3.53]	.24	−12.83 [−25.94–.29]	.055	−6.89 [−20.88–7.10]	.33	−8.24 [−20.22–3.74]	.18	**−.43** * **[−.83‐(−.04)]**	**.032** *
Providing sufficient portable play equipment^a^ (*n* = 266)	−8.62 [−17.67–.44]	.06	7.59 [−.56–15.73]	.07	−1.25 [−8.79–6.30]	.75	**9.46** * **[.88–18.05]**	**.031** *	2.35 [−10.35–15.05]	.72	3.84 [−9.97–17.66]	.58	**−15.47** * **[−27.00‐(−3.95)]**	**.009** *	−.35 [−.74–.04]	.08
Having a PA policy^a^ (*n* = 260)	**11.86** * **[1.64–22.07]**	**.023** *	6.36 [−2.96–15.67]	.18	−3.46 [−12.05–5.14]	.43	**−10.33** * **[−20.08–.58]**	**.038** *	−2.41 [−17.05–12.22]	.75	−2.21 [−17.82–13.39]	.78	−4.36 [−17.74–9.02]	.52	.03 [−.41–.48]	.88
Limiting the use of screen time (less is best)^a^ (*n* = 261)	**11.77** * **[5.22–18.32]**	**<.001** *	.17 [−5.86–6.20]	.95	.80 [−4.77–6.37]	.78	−3.96 [−10.31–2.39]	.22	.73 [−8.79–10.25]	.88	−1.12 [−11.27–9.04]	.83	3.17 [−5.50–11.83]	.47	**.44** * **[.16–.72]**	**.003** *

Abbreviations: HE, healthy eating; PA, physical activity.

^a^ Controlled for remoteness.

* Significant at *p* < .05.

Several statistically significant associations between service provider types and PA policy and practice implementation were observed. ‘Delivering teacher‐led, structured physical activity’ was implemented at a significantly lower extent in private not‐for‐profit community managed services (*β* = −9.62, 95% CI [−17.06‐(−2.18)], *p* = .011); in private not‐for‐profit other organisations (*β* = −12.78, 95% CI [−23.76‐(−1.80)], *p* = .023); and in state/territory and local government managed services (*β* = −14.41, 95% CI [−26.35‐(−2.47)], *p* = .018) than in private for‐profit services. ‘Having a PA policy’ and ‘scheduling more opportunities for outdoor free play’ were implemented at a significantly lower extent in private not‐for‐profit community managed services (*β* = −10.33, 95% CI [−20.08–(−.58)], *p* = .038, and *β* = −14.05, 95% CI [−21.52‐(−6.58)], *p* = <.001 respectively), but they provided sufficient portable play equipment at a significantly higher extent than private for‐profit services (*β* = 9.46, 95% CI [.88–18.05], *p* = .031). No statistically significant associations were found between the implementation of PA practices and socio‐economic status.

## 4 DISCUSSION

### 4.1 Key results and interpretation

This study reports on both the number of services implementing HE and PA policies and practice components *and* the extent of their implementation. Our findings show that while some HE and PA practices are implemented fully, other practices are implemented to a lesser extent. Importantly, education‐related HE and PA practices are implemented to a lower extent than other practices. Further, most services have either a HE or a PA policy, but certain types of service are more likely to implement these policies than others. Association analyses indicate that implementation support is particularly needed for services located in remote or regional areas, preschools and smaller services. Lastly, private for‐profit services report higher levels of implementation on most of the examined practices than other provider types. Implementation support should focus on practices with a lower level of implementation.

A key finding of the study is that the implementation of educational HE and PA practices such as ‘providing educator training on physical activity’, ‘providing opportunities for nutrition education to children’ and ‘supporting families to promote PA’ could be improved. Despite NSW ECEC services having free access to professional development and other educational resources since 2008,^37^ low percentages of services providing PA training to educators are observed, in line with a previous study in Australia.^52^ Training attendance is an important strategy that can increase the implementation of practices.^21^ For example, a study found that when family day care educators receive training, the odds of providing PA opportunities to children are higher than without training (OR = 1.85, *p* = <.001, CI [1.39–2.46]).^53^ Lack of engagement with professional development might be due to educators disliking being physically active or disliking a change in routine.^54^ There is a need to further explore factors that affect educators' access to and engagement with professional development and to examine how professional development can affect HE and PA policy and practice implementation. Low provision of nutritional education sessions for children may be due to competing activities in the curriculum, lack of time and resources as observed in services in Queensland, Australia.^55^ Including HE education in the routine can have positive behaviour change in children. There is a need to conduct more HE education interventions in preschools and to identify effective strategies to overcome barriers to the provision of HE education. In practice, targeted support could be strengthened through existing programs such as Munch & Move.

Encouragingly, over 90% of the respondents reported ‘having a HE policy’, contrasting with a previous Australian study in which 75% of participating services had a HE policy in 2016,^34^ and in the United States in which only 41% had a HE policy.^32^ Literature suggests that policy can improve implementation by providing a shared understanding of organisations' vision and regulation compliance. However, having a policy does not necessarily mean all practices recommended in it will be implemented, and a lack of policy might be not causing low practice implementation. For example, educators in a service might be role modelling health behaviours during mealtimes (practice), but service may not have a HE policy. Barriers to implementation of HE policies include lack of inclusiveness (e.g. omission of certain age groups), being too technical, lacking clear guidance for educators^56^ and lacking comprehensiveness.^57^, ^58^ Further research is needed to investigate the effectiveness of HE policy implementation specifically *and* the provision of implementation support on service and educator HE practices uptake. The Play Active PA Policy study conducted in Western Australia and the Childcare PA Policy in Canada, for example, found an increase in policy practices uptake^59^ and an increase in child light PA levels^30^ when implementation support was provided to deliver components of the policy. Future research examining policy implementation should also consider using validated measures such as the WellCCAT to support assessment.

Association analyses suggest that implementation support for several practices is particularly needed for services located in remote or regional areas, preschools and smaller services. These practices include ‘having a PA policy’, ‘limiting the use of screen time’, ‘providing sufficient portable play equipment’, ‘providing educator training on PA’, ‘engaging families in activities to increase child PA’ and ‘encouraging supportive feeding practices’. Low implementation of HE/PA practices in preschools might be potentially due to differences in organisation structure as preschools differ from long day care services in hours of operation, number of children enrolled and staff numbers. Also, they may have competing curriculum priorities such as school readiness, or may lack financial resources to attend training, cover teacher relief costs and purchase play equipment. This may contribute to an increased inability to deliver PA practices. Services located in regional/remote areas may lack human resources to ensure adequate supervision of children or may lack knowledge or skills on how to engage with families to increase PA or encourage supportive feeding practices. Future research exploring the factors that affect the implementation of PA policies and practices in different organisational characteristics and structures, service locations, support systems (i.e. trainings) and community‐level factors^60^ is needed.

Our findings identified that, on average, private for‐profit services scored significantly higher than other provider types in implementation of several of the HE and PA policies and practices. This finding may be due to the differing governance structures, with a higher percentage of private for‐profit services attached to large providers (35%)^61^ which may facilitate the standardisation and implementation of HE and PA policies and practices. These associations should be interpreted with caution as the sample size of the provider type subgroups was relatively small. Future research considering governance of services should be considered to better understand the impact of different provider types on implementation of HE and PA practices.

### 4.2 Strengths and limitations

Key strengths of the study include a large sample size, inclusion of services located across the geographically and socio‐economically diverse state of NSW and a detailed reporting of implementation levels of HE and PA practice components. Such information provides a comprehensive understanding into the extent (e.g. the frequency and amount) to which policies and practices are being implemented in centre‐based ECEC services to achieve population changes in PA and HE behaviours.^62^ This detail may support tailoring and focussing of future research and implementation support efforts to those specific components of HE and PA policies and practices difficult to implement. Nonetheless, this study also has limitations that should be considered. First, components within each policy and practice were weighted equally when calculating the mean proportion of implementation. While it may be that some components yield a greater effect on child PA and HE and should therefore be prioritised when defining thresholds for implementation, to our knowledge, there is limited evidence available of the isolated effects of discrete components to enable such weighting, due to the multicomponent nature of HE and PA interventions in the setting.^29^ As response options varied for each component, and the number of components varied for each policy and practice, results should be interpreted with caution. Second, we assessed the implementation of HE and PA policies and practices using a measure based on validated surveys either by telephone or via online. While self‐reporting tools have been shown to accurately report the implementation of HE and PA practices,^43^ post hoc tests revealed that participants who responded via telephone implemented significantly more HE and PA practices than those who responded online. This may be due to social desirability bias,^41^ whereby respondents may answer questions in a manner that they think will be viewed favourably by the caller. As such, this study may have resulted in an overestimation of the implementation of practices.^45^ This survey was completed by either director/assistant director (40%) or nominated supervisor (51%) who supervise overall service operations. It should be acknowledged that while differences may exist between their responses, participants might have multiple roles (not captured in study) and therefore it is difficult to observe differences through a quantitative survey. Further qualitative research may be beneficial to identify how these roles perceive and influence policy and practice implementation. Third, this study was cross‐sectional in nature, therefore we cannot infer causation. Further, we did not measure implementation at different time points and therefore, we relied on participants' memory to assess the frequency and duration of the implementation of practices. Therefore, we encourage further research including qualitative research design to explore in more depth factors impacting implementation, along with longitudinal studies to measure sustainability of policy and practice over time. Fourth, while this study measured the implementation of HE and PA policies and practices in detail, they were measured independently. The focus of the study was not to understand the interaction between HE and PA policies and practices, nor a comparison of the implementation of both sets of practices in each service. Further research should examine the implementation of both HE and PA practices within each service to better understand the overall quality of the health promoting environment of ECEC services. Lastly, this study was conducted during the COVID‐19 pandemic and local lockdowns and while some regions in NSW were affected by severe floods, which might have impacted on consent rates and survey responses. The implementation of several policies and practices may have been affected due to challenging staffing arrangements, services temporally closed, educators' level of stress and uncertainty regarding the changing COVID‐19 environment.^49^, ^63^

## 5 CONCLUSIONS

This study provides detailed findings suggesting that while many HE and PA policies and practices and their components are being implemented, there is still substantial scope for improvement regarding the implementation of evidence‐based HE and PA policies and practices in the ECEC setting in NSW. Examining the barriers and enablers to implementation of components of practices and overall policy and practice implementation may be of benefit to facilitate identification and tailoring of evidence‐based strategies to support implementation. Future implementation support efforts and research should be prioritised where there is low implementation.

## FUNDING INFORMATION

The study was funded by a NHMRC Centre for Research Excellence—National Centre of Implementation Science (NCOIS) grant (CIA: Wolfenden, APP1153479). This research was supported by a PhD scholarship (Australian research training program) linked to the NCOIS. AG is supported by a Heart Foundation Postdoctoral Fellowship (102518). SY is supported by a Heart Foundation Future Leader Fellowship (106654). RKH is supporting by a NHMRC Early Career Fellowship (APP1160419).

## CONFLICT OF INTEREST STATEMENT

The authors declare that they have no competing interests.

## ETHICS STATEMENT

This research received approval from Hunter New England Human Research Ethics Committee (06/07/26/4.04 2019/ETH12353) and the University of Newcastle Human Research Ethics Committee (H‐2008‐0343).

## Data Availability

The data that support the findings of this study are available from the corresponding author upon reasonable request.
